# Speech Impairment in Early Parkinson’s Disease Is Associated with Nigrostriatal Dopaminergic Dysfunction

**DOI:** 10.3390/jcm15031006

**Published:** 2026-01-27

**Authors:** Sotirios Polychronis, Grigorios Nasios, Efthimios Dardiotis, Rayo Akande, Gennaro Pagano

**Affiliations:** 1 Department of Speech & Language Therapy, School of Health Sciences, University of Ioannina, 45500 Ioannina, Greece; 2 Department of Medicine, University of Thessaly, 41500 Larissa, Greece; edar@med.uth.gr; 3 Department of Neurology, University Hospital of Larissa, 41110 Larissa, Greece; 4 F. Hoffmann-La Roche Ltd., Neuroscience and Rare Diseases, Roche Pharma Research and Early Development, 4058 Basel, Switzerland; rayo.akande@roche.com (R.A.); gennaro.pagano@roche.com (G.P.)

**Keywords:** Parkinson’s disease, hypokinetic dysarthria, speech difficulties, dopaminergic deficit, [^123^I]FP-CIT, putamen, basal ganglia, cerebrospinal fluid biomarkers, levodopa, motor impairment

## Abstract

**Background/Objectives:** Speech difficulties are an early and disabling manifestation of Parkinson’s disease (PD), affecting communication and quality of life. This study aimed to examine demographic, clinical, dopaminergic imaging and cerebrospinal fluid (CSF) correlates of speech difficulties in early PD, comparing treatment-naïve and levodopa-treated patients. **Methods:** A cross-sectional analysis was conducted using data from the Parkinson’s Progression Markers Initiative (PPMI). The sample included 376 treatment-naïve and 133 levodopa-treated early PD participants. Speech difficulties were defined by Movement Disorder Society—Unified Parkinson’s Disease Rating Scale (MDS-UPDRS) Part III, with Item 3.1 ≥ 1. Group comparisons and binary logistic regression identified predictors among demographic, clinical, dopaminergic and CSF biomarker variables, including [^123^I]FP-CIT specific binding ratios (SBRs). All analyses were cross-sectional, and findings reflect associative relationships rather than treatment effects or causal mechanisms. **Results:** Speech difficulties were present in 44% of treatment-naïve and 57% of levodopa-treated participants. In both cohorts, higher MDS-UPDRS Part III ON scores—reflecting greater motor severity—and lower mean putamen SBR values were significant independent predictors of speech impairment. Age was an additional predictor in the treatment-naïve group. No significant differences were found in CSF biomarkers (α-synuclein, amyloid-β, tau, phosphorylated tau). These findings indicate that striatal dopaminergic loss, particularly in the putamen, and motor dysfunction relate to early PD-related speech difficulties, whereas CSF neurodegeneration markers do not differentiate affected patients. **Conclusions:** Speech difficulties in early PD are primarily linked to dopaminergic and motor dysfunction rather than global neurodegenerative biomarker changes. Longitudinal and multimodal studies integrating acoustic, neuroimaging, and cognitive measures are warranted to elucidate the neural basis of speech decline and inform targeted interventions.

## 1. Introduction

Speech difficulties are a common and disabling feature of Parkinson’s disease (PD), affecting up to 90% of patients as the condition progresses and often emerging early, even before motor diagnosis [1,2,3]. Around 40% of newly diagnosed, untreated patients already report speech problems, highlighting their potential as early clinical indicators of PD [4,5,6]. These impairments limit communication, social participation, and quality of life, underscoring their clinical significance [6]. The speech disorder most often associated with PD is hypokinetic dysarthria, caused by basal ganglia dysfunction and affecting respiration, phonation, articulation, and prosody [7,8,9]. Typical features include reduced loudness, monopitch, imprecise consonants, and variable speech rate [7]. Such abnormalities reflect underlying motor deficits—rigidity, bradykinesia, and tremor—that compromise articulatory precision and vocal control [10,11]. However, speech impairment in PD is multifactorial, also involving sensory and cognitive components, altered auditory feedback, and neurotransmitter imbalances beyond dopaminergic loss [12,13,14]. Neuroimaging studies reveal that speech difficulties are linked to abnormal activity and connectivity across cortico-basal ganglia–cerebellar networks [15,16,17,18], while cerebrospinal fluid (CSF) biomarkers such as reduced amyloid-β, elevated tau, and decreased α-synuclein provide complementary insights into underlying neurodegeneration and cognitive risk [19,20,21,22]. Together, these findings suggest that speech changes may mirror early neural and biochemical alterations in PD.

The present study aims to describe and compare the demographic, clinical, CSF biomarker, and presynaptic dopaminergic characteristics of early PD patients with and without speech difficulties, in both treatment-naïve and early levodopa-treated stages. Speech difficulties were measured using the examiner-rated speech item of the MDS-UPDRS Part III. Understanding these associations may clarify the mechanisms underlying PD-related speech changes. Importantly, this study adopts a cross-sectional design, and comparisons between treatment-naïve and levodopa-treated patients reflect group-level associations rather than longitudinal progression or treatment effects.

## 2. Materials and Methods

### 2.1. Study Design and Data Source

This study was a cross-sectional analysis using data from the Parkinson’s Progression Markers Initiative (PPMI), a large, multicentre, observational cohort designed to identify and validate biomarkers of PD. Access to the dataset was granted following approval by the PPMI Data Access Committee. The PPMI applies standardised operating procedures for participant evaluation, data collection, biospecimen handling, and imaging, ensuring methodological consistency across sites. Comprehensive study information is publicly available at https://www.ppmi-info.org/study-design (accessed on 29 January 2024).

### 2.2. Participants

Data were extracted from the PPMI curated dataset (version 2024-01-29), including participants with sporadic PD. Inclusion criteria comprised: diagnosis within the previous two years, age ≥ 30 years at diagnosis, presence of at least two cardinal motor features (resting tremor, bradykinesia, or rigidity) with tremor or bradykinesia required, Hoehn and Yahr stage I–II at baseline, and evidence of presynaptic dopaminergic deficit on imaging. Exclusion criteria included use of PD-specific medication or agents interfering with dopamine transporter imaging within six months of enrolment, and medical contraindications to lumbar puncture (e.g., spinal abnormalities, coagulopathy).

Two independent groups were defined. The early PD treatment-naïve group included participants assessed at baseline before initiating any antiparkinsonian therapy. The early PD levodopa-treated group included participants assessed at Year 2 (Visit 6) after initiating levodopa. The groups comprised distinct individuals and were analysed separately.

### 2.3. Clinical Assessment

Clinical evaluation included the MDS-UPDRS Parts I–IV, the Montreal Cognitive Assessment (MoCA), and the Hoehn and Yahr scale. Speech impairment was assessed using item 3.1 of the MDS-UPDRS Part III (Motor Examination), an examiner-rated measure of overall speech clarity and intelligibility during clinical assessment. This item provides a global index of speech impairment but does not distinguish between individual speech subsystems (e.g., phonatory, articulatory, or prosodic deficits) and does not capture patient-reported communicative participation. Motor severity was assessed using MDS-UPDRS Part III (excluding Item 3.1, Speech), and functional independence using MDS-UPDRS Part II (excluding Item 2.3, Chewing and Swallowing).

### 2.4. Dopaminergic Imaging

Single-photon emission computed tomography (SPECT) imaging was performed four hours (±30 min) after administration of 3–5 mCi (111–185 MBq) [^123^I]FP-CIT (DaTscan™) or 3.5 h (±30 min) after 25 mCi (925 MBq) ^99m^Tc-TRODAT-1. Imaging adhered to the PPMI SPECT Technical Operations Manual. Data were acquired in a 128 × 128 matrix using a step-and-shoot protocol (120 projections over 360°, 30 s per projection) and energy windows centred at 159 keV (±10%) for DaTscan™ or 140 keV (±10%) for TRODAT-1.

For quantitative analysis, reconstructed image volumes were spatially normalised to a standardised Ioflupane template. The eight axial slices best depicting the striatum were summed, and standardised volumes of interest (VOIs) were applied to the caudate and putamen bilaterally, using the occipital cortex as a reference region. Specific binding ratios (SBRs) were calculated as (striatum/occipital cortex − 1), providing an approximation of binding potential (BPND) at tracer equilibrium.

### 2.5. Cerebrospinal Fluid Biomarker Analysis

Cerebrospinal fluid samples were collected by lumbar puncture at baseline and follow-up visits according to PPMI biospecimen protocols. Quantification of amyloid-β, α-synuclein, total tau, and phosphorylated tau concentrations was performed using validated immunoassays.

### 2.6. Statistical Analysis

#### 2.6.1. Study Population and Speech Difficulty Classification

Statistical analyses were conducted using SPSS (Version 29; IBM Corp., Armonk, NY, USA). The final sample included 376 early PD treatment-naïve participants (167 with and 209 without speech difficulties) and 133 early PD levodopa-treated participants (76 with and 57 without speech difficulties). Speech difficulties were defined using MDS-UPDRS Part III, Item 3.1 (Speech), with scores ≥1 indicating impairment.

#### 2.6.2. Group Comparisons

Comparisons were performed separately for treatment-naïve and levodopa-treated groups. Normality and variance homogeneity were evaluated using descriptive statistics and Levene’s test. Independent *t*-tests compared demographic, clinical, and neuroimaging variables between subgroups. Missing data were handled with pairwise exclusion, and sensitivity analyses were performed when >20% of values were missing. Given the exploratory design, multiple comparison corrections were not applied; instead, results were interpreted in the context of effect sizes (Cohen’s *d*) and clinical relevance. Variables with *p* < 0.05 in univariate analyses were entered into multivariate models.

#### 2.6.3. Multivariate Analysis

Binary logistic regression models were constructed to identify independent predictors of speech difficulties in treatment-naïve and levodopa-treated participants. Predictor variables were selected based on univariate significance and theoretical relevance. Multicollinearity was assessed using correlation matrices (*r* > 0.7) and the Variance Inflation Factor (VIF < 10).

To minimise redundancy and improve model stability, correlated predictors were reduced to representative variables. MDS-UPDRS Part III ON was retained as the most clinically relevant measure of motor impairment, replacing MDS-UPDRS Total and MDS-UPDRS Total ON (which were highly correlated, *r* > 0.8). Among neuroimaging parameters, mean putamen was selected as the primary dopaminergic marker due to strong correlations with mean caudate and mean striatum (*r* > 0.7). The same multicollinearity assessment and variable-selection procedures were applied to both the treatment-naïve and levodopa-treated regression models.

Model fit was evaluated using the Hosmer–Lemeshow test and Nagelkerke’s R^2^. Odds ratios (OR) and 95% confidence intervals (CI) were reported for all predictors.

### 2.7. Ethical Considerations

This research involved secondary analysis of de-identified data from the PPMI database (ClinicalTrials.gov Identifier: NCT01141023). The PPMI study was approved by the Institutional Review Boards of all participating centres and conducted in accordance with the Declaration of Helsinki. Written informed consent was obtained from all participants prior to enrolment. The present analysis was exempt from additional ethical review, as it used publicly available, anonymized data. Reporting followed the STROBE (Strengthening the Reporting of Observational Studies in Epidemiology) guidelines [23].

## 3. Results

All analyses were cross-sectional. Comparisons between treatment-naïve and levodopa-treated groups were conducted at the group level and do not reflect within-subject longitudinal change or treatment responsiveness.

### 3.1. Speech Difficulties in Early Parkinson’s Disease Treatment-Naïve Patients

#### 3.1.1. Group Comparisons

##### Demographic Characteristics

In early treatment-naïve PD patients, 167 participants with speech difficulties were compared to 209 participants without speech difficulties. Patients with speech difficulties were significantly older (mean ± SD: 63.78 ± 8.8 years) than those without (60.33 ± 9.9 years; *p* < 0.001). The sex distribution also differed significantly, with a male-to-female ratio of 0.71 in the speech difficulties group versus 0.61 in the group without speech difficulties (*p* = 0.04). Disease duration did not differ between groups (6.63 ± 6.1 vs. 6.65 ± 6.8 years; *p* = 0.98) (Table 1).

##### Clinical Characteristics

**Figure 1:**
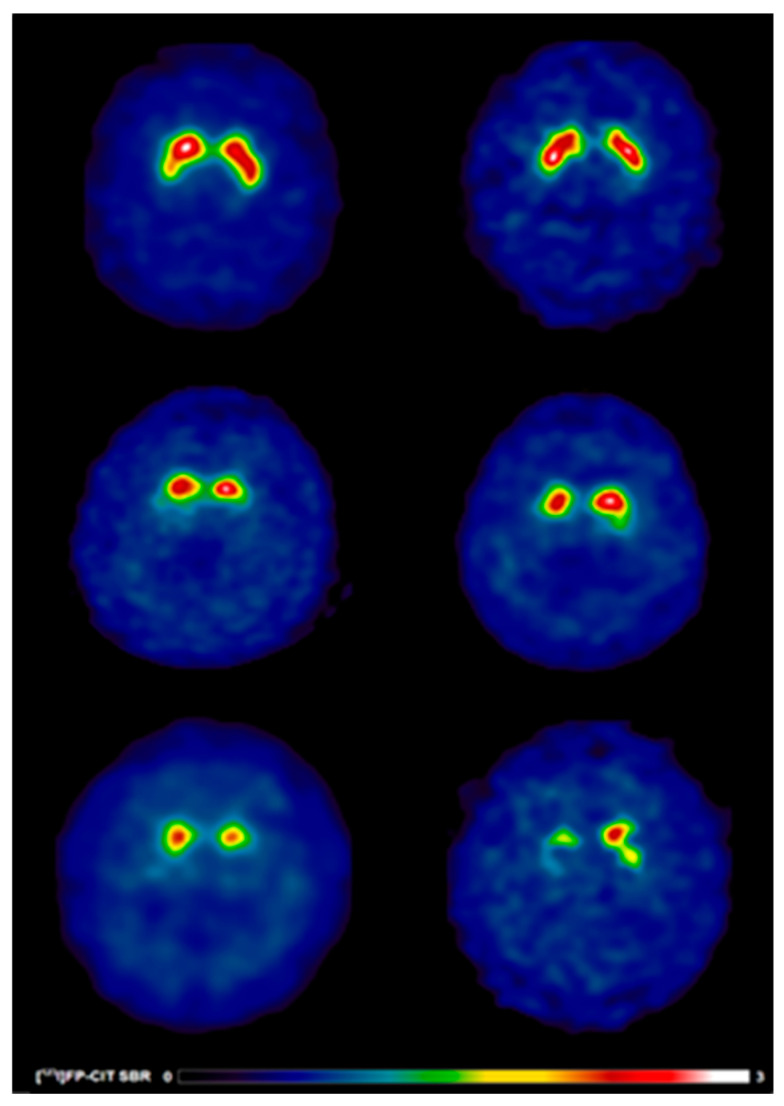
[^123^I]FP-CIT SPECT images in early PD patients with and without speech difficulties. Reduced [^123^I]FP-CIT binding reflects presynaptic nigrostriatal dopaminergic degeneration and should not be interpreted as an index of treatment responsiveness or pharmacological effect. Warmer colours indicate higher dopamine transporter availability, whereas cooler colours indicate reduced presynaptic dopaminergic binding. Anatomical regions of interest include the caudate nucleus and putamen bilaterally. (**Top**) A 63-year-old healthy control male (left) showing typical [^123^I]FP-CIT specific binding ratios in the caudate (SBR: 3.03) and putamen (SBR: 2.26) and a 69-year-old healthy control female (right) showing typical [^123^I]FP-CIT specific binding ratios in the caudate (SBR: 3.21) and putamen (SBR: 2.79). (**Middle**) A 63-year-old male (left) without speech difficulties exhibiting slight dopaminergic deficits as reflected by [^123^I]FP-CIT specific binding ratios in the caudate (SBR: 1.98) and putamen (SBR: 0.52) and a 69-year-old female (right) without speech difficulties exhibiting slight dopaminergic deficits as reflected by [^123^I]FP-CIT specific binding ratios in the caudate (SBR: 2.45) and putamen (SBR: 0.79). (**Bottom**) A 63-year-old male (left) with speech difficulties demonstrating larger striatal dopaminergic deficits as reflected by [^123^I]FP-CIT specific binding ratios in the caudate (SBR: 1.16) and putamen (SBR: 0.37) and a 69-year-old male (right) with speech difficulties demonstrating larger striatal dopaminergic deficits as reflected by [^123^I]FP-CIT specific binding ratios in the caudate (SBR: 1.33) and putamen (SBR: 0.77).

Participants with speech difficulties exhibited a higher burden of non-motor symptoms compared to those without speech difficulties (MDS-UPDRS Part I: 6.1 vs. 5.0; *p* = 0.01) (Figure 1). They also demonstrated greater motor symptom severity in both the OFF-state (MDS-UPDRS Part II: 7.2 vs. 4.9, *p* < 0.001; MDS-UPDRS Part III: 24.1 vs. 18.5, *p* < 0.001) and ON-state (MDS-UPDRS Part III: 24.1 vs. 18.5, *p* < 0.001; Hoehn & Yahr: 1.7 vs. 1.5, *p* < 0.001). Overall disease burden was higher in patients with speech difficulties in the OFF-state (MDS-UPDRS Total: 37.4 vs. 28.5; *p* < 0.001) and ON-state (37.4 vs. 28.5; *p* < 0.001). Cognitive performance, assessed with the MoCA, did not significantly differ between groups (27.0 vs. 27.3; *p* = 0.20) (Table 1).

##### CSF Biomarkers

No significant differences in CSF biomarker concentrations were observed between participants with and without speech difficulties (Table 1).

##### [^123^I]FP-CIT SBR Values

Analysis of [^123^I]FP-CIT SBR values revealed significant reductions in PD patients with speech difficulties compared to those without. Specifically, caudate binding was lower bilaterally (1.8 vs. 2.1; *p* < 0.001), as well as contralaterally (1.7 vs. 1.9; *p* = 0.002) and ipsilaterally (2.0 vs. 2.2; *p* < 0.001) relative to the most affected side. Putamen SBR values showed similar trends, with reductions in bilateral (0.7 vs. 0.87; *p* < 0.001), contralateral (0.6 vs. 0.7; *p* < 0.001), and ipsilateral (0.8 vs. 1.0; *p* < 0.001) regions. Striatal binding was also significantly lower in patients with speech difficulties across bilateral (1.3 vs. 1.5; *p* < 0.001), contralateral (2.3 vs. 2.6; *p* < 0.001), and ipsilateral (2.8 vs. 3.3; *p* < 0.001) measures (Table 1; Figure 1).

#### 3.1.2. Multivariate Binary Logistic Regression Analysis

The final logistic regression model included age, sex, MDS-UPDRS Part III ON, Hoehn and Yahr ON, and mean putamen as independent predictors (Table 2). The model was statistically significant, χ^2^(5) = 61.420, *p* < 0.001, indicating that the combined predictors reliably differentiated participants with and without speech difficulties. Nagelkerke’s R^2^ = 0.202 suggested that the model explained 20.2% of the variance in speech difficulties, while the non-significant Hosmer–Lemeshow test (*p* = 0.588) confirmed good model fit.

Higher MDS-UPDRS Part III ON scores were significantly associated with increased odds of speech difficulties (OR = 1.074, *p* < 0.001), indicating that greater motor impairment strongly contributes to speech problems. Lower mean putamen values also predicted speech difficulties (OR = 0.198, *p* < 0.001), highlighting the role of striatal neurodegeneration. Age was a modest but significant predictor (OR = 1.028, *p* = 0.028), with each additional year increasing the likelihood of speech difficulties by approximately 2.8%.

Sex (*p* = 0.104) and Hoehn and Yahr ON (*p* = 0.393) were not significant in the final model, suggesting that their univariate associations were driven by shared variance with motor severity and neurodegenerative markers. Overall, these findings demonstrate that age, motor impairment, and putaminal dopaminergic deficits are key determinants of speech difficulties in early treatment-naïve PD.

### 3.2. Speech Difficulties in Early Parkinson’s Disease Treatment-Naïve Patients

#### 3.2.1. Group Comparisons

##### Demographic Characteristics

In early PD patients receiving levodopa, the group with speech difficulties (N = 76) was compared to the group without speech difficulties (N = 57). No significant differences were observed in mean age, sex ratio, or disease duration between the two groups (Table 3).

##### Clinical Characteristics

Levodopa-treated PD patients with speech difficulties exhibited significantly greater motor symptom severity compared to those without speech difficulties. Specifically, they had higher UPDRS Part II scores (8.7 vs. 6.2, *p* = 0.003), higher UPDRS Part III scores in the OFF-state (29.3 vs. 21.9, *p* < 0.001) and ON-state (24.0 vs. 17.0, *p* < 0.001), and higher Hoehn and Yahr scores (1.8 vs. 1.6, *p* = 0.045; Table 3). Overall disease burden was also elevated in patients with speech difficulties on both OFF and ON medication (UPDRS Total OFF-state: 45.6 vs. 34.0, *p* < 0.001; UPDRS Total ON-state: 40.0 vs. 29.6, *p* < 0.001; Table 3).

No significant differences were observed between the groups in cognitive performance (MoCA), motor complications (MDS-UPDRS Part IV), or non-motor symptom burden (MDS-UPDRS Part I) (Table 3).

##### CSF Biomarkers

Analysis of cerebrospinal fluid biomarkers revealed no significant differences between patients with and without speech difficulties (Table 3).

##### [^123^I]FP-CIT SBR

**Figure 2:**
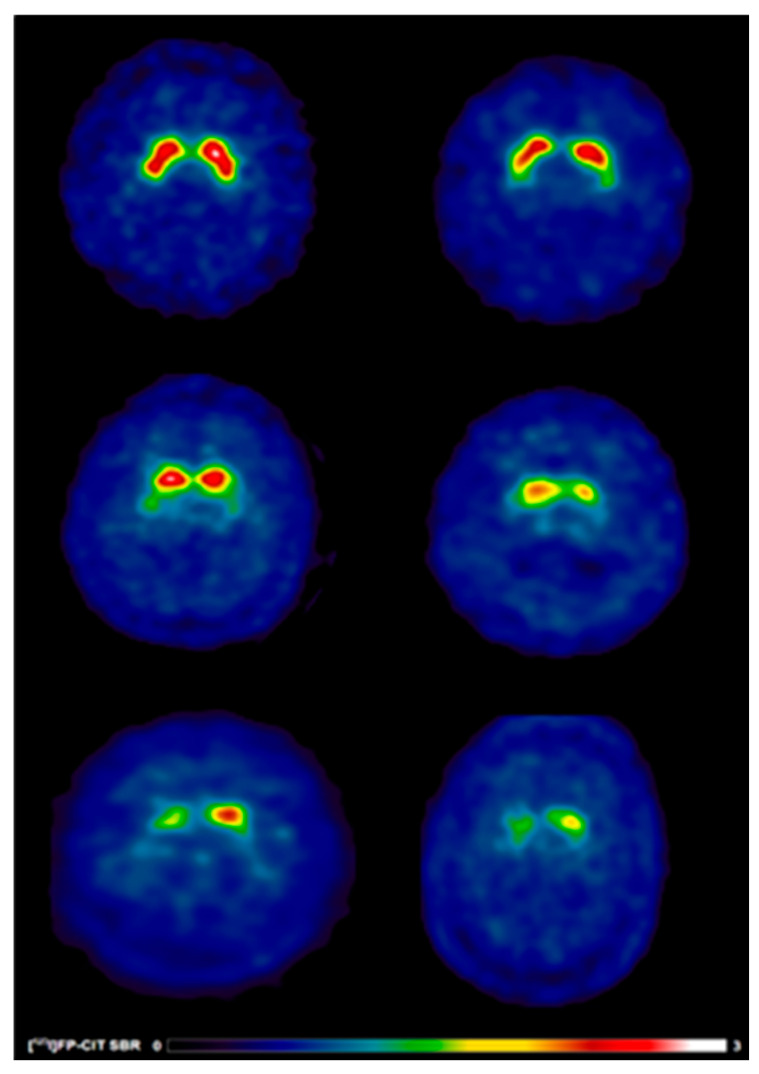
[^123^I]FP-CIT SPECT images in early treated PD patients with and without speech difficulties. Reduced [^123^I]FP-CIT binding reflects presynaptic nigrostriatal dopaminergic degeneration and should not be interpreted as an index of treatment responsiveness or pharmacological effect. Warmer colours indicate higher dopamine transporter availability, whereas cooler colours indicate reduced presynaptic dopaminergic binding. Anatomical regions of interest include the caudate nucleus and putamen bilaterally. (**Top**) A 65-year-old healthy control male (left) showing typical [^123^I]FP-CIT specific binding ratios in the caudate (SBR: 4.15) and putamen (SBR: 2.89) and a 69-year-old healthy control male (right) showing typical [^123^I]FP-CIT specific binding ratios in the caudate (SBR: 2.75) and putamen (SBR: 1.85). (**Middle**) A 65-year-old male (left) without speech difficulties exhibiting slight dopaminergic deficits as reflected by [^123^I]FP-CIT specific binding ratios in the caudate (SBR: 2.38) and putamen (SBR: 0.99) and a 69-year-old female (right) without speech difficulties exhibiting slight dopaminergic deficits as reflected by [^123^I]FP-CIT specific binding ratios in the caudate (SBR: 1.4) and putamen (SBR: 0.54). (**Bottom**) A 65-year-old male (left) with speech difficulties demonstrating larger striatal dopaminergic deficits as reflected by [^123^I]FP-CIT specific binding ratios in the caudate (SBR: 0.92) and putamen (SBR: 0.45) and a 69-year-old male (right) with speech difficulties demonstrating larger striatal dopaminergic deficits as reflected by [^123^I]FP-CIT specific binding ratios in the caudate (SBR: 1.36) and putamen (SBR: 0.71).

Significant differences in striatal [^123^I]FP-CIT SBR values were observed between groups. Patients with speech difficulties exhibited reduced SBR values in the caudate: contralateral (1.4 vs. 1.6, *p* = 0.02), ipsilateral (1.7 vs. 1.9, *p* = 0.02), and bilateral (1.5 vs. 1.7, *p* = 0.01). Putamen SBR values followed similar trends, with reductions in contralateral (0.5 vs. 0.6, *p* = 0.003), ipsilateral (0.7 vs. 0.8, *p* = 0.008), and bilateral (0.6 vs. 0.7, *p* = 0.002) regions. Striatal SBR was also significantly lower in patients with speech difficulties: contralateral (1.9 vs. 2.2, *p* = 0.008), ipsilateral (2.3 vs. 2.7, *p* = 0.01), and bilateral (1.1 vs. 1.2, *p* = 0.007) (Table 3; Figure 2).

#### 3.2.2. Multivariate Binary Logistic Regression Analysis

The final logistic regression model included MDS-UPDRS Part III ON and mean putamen as independent predictors (Table 4). The model was statistically significant, χ^2^(2) = 22.907, *p* < 0.001, indicating that these variables jointly distinguished participants with and without speech difficulties. Nagelkerke’s R^2^ = 0.231 showed that the model explained 23.1% of the variance in speech difficulties, while the non-significant Hosmer–Lemeshow test (*p* = 0.809) confirmed an adequate model fit.

Higher MDS-UPDRS Part III ON scores were significantly associated with increased odds of speech difficulties (OR = 1.075, *p* = 0.002), indicating that greater motor impairment in the ON state contributes to the likelihood of speech problems. Lower mean putamen values were also significant predictors (OR = 0.082, *p* = 0.016), highlighting the contribution of striatal dopaminergic deficits.

MDS-UPDRS Part II and Hoehn and Yahr ON, although significant in univariate analyses, were not retained in the final model, suggesting that their effects were mediated by stronger predictors such as MDS-UPDRS Part III ON and mean putamen. Overall, these findings confirm the combined influence of motor dysfunction and neurodegeneration on the manifestation of speech difficulties in early levodopa-treated PD patients.

## 4. Discussion

All findings should be interpreted as associative relationships derived from cross-sectional data and do not imply causal mechanisms or treatment effects.

### 4.1. Demographic and Clinical Correlates of Speech Difficulties in Early Parkinson’s Disease

Speech difficulties were prevalent in both treatment-naïve and levodopa-treated patients with early PD and were consistently associated with greater overall motor burden. Examination of demographic and clinical characteristics provides important context for understanding the clinical phenotype in which speech impairment emerges and persists during early disease stages.

Speech difficulties can appear even at the de novo stage, consistent with prior work in early PD [4]. In treatment-naïve patients, age was a significant predictor, supporting the view that advancing age reduces neuromuscular control and neural plasticity and increases vulnerability to neurodegeneration, thereby amplifying speech difficulties [24,25]. Reduced capacity for compensatory recruitment across cortico-basal ganglia circuits may further burden speech motor control with age [26]. Although one de novo cohort found no age difference between those with and without speech difficulties, a higher prevalence among akinetic-rigid phenotypes was observed, suggesting an interaction with motor subtype [4].

Sex differences (more males among those with speech difficulties) were observed descriptively, but sex did not remain an independent predictor after adjustment, implying broadly comparable risk between men and women when motor severity and neurodegeneration are controlled. Small male–female contrasts reported elsewhere tend to diminish after accounting for disease severity [27]. Disease duration did not differ between groups, reinforcing that early speech involvement may relate more to age and motor phenotype than to elapsed time since diagnosis [4].

In early levodopa-treated patients, speech difficulties remained prevalent, but age, sex ratio, and disease duration did not differ between those with and without speech difficulties, suggesting that factors beyond simple demographics—such as treatment effects, motor phenotype, and underlying dopaminergic degeneration—may be more influential at this stage [28]. While ageing continues to shape neuromuscular reserve, dopaminergic therapy may partially mask demographic contrasts evident pre-treatment, even as speech problems persist.

The findings of this study indicate that speech difficulties in early PD are strongly associated with greater overall motor burden, as reflected by higher MDS-UPDRS scores across both treatment-naïve and levodopa-treated patients. This pattern reinforces the view that speech difficulties in PD are primarily a motor phenomenon rather than a secondary feature of disease progression or cognitive decline. Elevated MDS-UPDRS Part III ON scores in particular emerged as the strongest predictor of speech difficulties, underscoring the central contribution of bradykinesia, rigidity, and reduced coordination in respiratory and articulatory musculature to hypokinetic dysarthria [8,24].

This relationship between motor severity and speech dysfunction aligns closely with previous research showing that axial symptoms—rather than tremor—are the primary motor correlates of dysarthria in PD [25]. Polychronis et al., 2019 also demonstrated that the akinetic-rigid phenotype presents a markedly higher prevalence of speech difficulties than the tremor-dominant form, suggesting that the degree of bradykinesia plays a determining role [4]. The absence of a significant relationship between Hoehn and Yahr staging and speech difficulties in the current analysis further supports the notion that dysarthria is not simply a by-product of overall disease stage but reflects localised degeneration in circuits mediating motor-speech control [26].

Beyond motor dysfunction, the current findings and prior work highlight the contribution of non-motor and cognitive mechanisms to speech decline. Patients with speech difficulties often present more pronounced autonomic disturbances, increased sleepiness, and REM sleep behaviour disorder symptoms, consistent with prior evidence linking these features to speech difficulties in early PD [4]. Although cognitive scores (MoCA) did not differ significantly between groups, earlier research indicates that deficits in temporal processing, attention, and executive function may exacerbate speech disfluency and lexical retrieval difficulties, particularly in the context of parallel progression of axial and motor symptoms [29,30,31,32,33]. Neuroimaging findings have similarly revealed reduced fronto-striatal and parietal connectivity, implicating disrupted phonological planning and self-monitoring [34,35,36]. Moreover, progressive voice-frequency variability and reduced rhythmicity have been proposed as early markers of cognitive decline [37,38], which may explain the long-term communicative difficulties observed in some PD subgroups.

Functionally, the persistence of speech difficulties despite mild disease stages and preserved cognition has substantial communicative consequences. PD patients frequently report conversational breakdowns characterised by reduced loudness, prolonged pauses, and vague or imprecise language, contributing to social withdrawal and reduced quality of life [38,39,40,41,42,43]. In addition, deficits in social cognition—such as impaired recognition of prosodic and emotional cues, reduced facial expressivity, and fewer spontaneous gestures—further constrain effective interpersonal communication [44,45,46,47,48].

### 4.2. Striatal Dopaminergic Imaging Correlates of Speech Difficulties

Beyond clinical motor severity, dopaminergic imaging findings provided converging evidence linking speech difficulties to striatal neurodegeneration, particularly within the putamen.

The present study demonstrates that speech difficulties in early PD are closely linked to dopaminergic deficits within striatal structures, particularly the putamen. Both treatment-naïve and levodopa-treated patients with speech difficulties exhibited significantly lower [^123^I]FP-CIT SBR compared with those without speech difficulties, supporting the view that reduced presynaptic dopaminergic function contributes to early speech motor dysfunction. Regression analyses further confirmed that lower mean putamen values were strong predictors of speech difficulties, highlighting the critical role of basal ganglia integrity in regulating articulation, timing, and fluency [4,49,50].

These results align with neuroimaging evidence indicating that speech difficulties in PD reflect disrupted connectivity across the cortico-basal ganglia-cerebellar network. functional Magnetic Resonance Imaging (fMRI) and Positron emission tomography (PET) studies have revealed abnormal activation within the orofacial motor cortex, supplementary motor area (SMA), and cerebellum, together with compensatory overactivation of premotor and prefrontal regions [15,16,17,18,51,52]. Such alterations likely reflect the brain’s attempt to maintain motor-speech output despite reduced dopamine availability. Notably, Elfmarková et al. (2016) demonstrated that levodopa modulates connectivity between the caudate and dorsolateral prefrontal cortex during prosody tasks [18], whereas Skodda et al. (2011) found no significant change in speech rate following levodopa intake, underscoring the incomplete responsiveness of speech control circuits to dopaminergic therapy [53].

Consistent with this, our findings support the notion that while dopamine depletion contributes substantially to speech difficulties, dopaminergic therapy alone is insufficient to normalise speech performance. These results indicate that speech motor control involves a broader network of cortical, subcortical, and cerebellar regions beyond the basal ganglia [54].

Neuroimaging evidence offers insight into these compensatory processes. Arnold et al. (2013) demonstrated that early-stage PD patients exhibit reduced connectivity between the caudate nucleus (CN) and prefrontal areas—including the inferior frontal gyrus, dorsolateral prefrontal cortex, and SMA—irrespective of medication status [54]. This hypo-connectivity may reflect impaired cognitive preparation for speech, leading to reduced vocal intensity (hypophonia). Dopamine intake appears to facilitate partial compensation by enhancing connectivity between the dorsal premotor cortex and SMA, suggesting that levodopa promotes alternative pathways supporting motor initiation [15,54,55]. Nonetheless, persistent deficits in auditory–motor integration and self-monitoring of speech intensity indicate that some components of the speech network remain resistant to dopaminergic modulation [54,56].

Recent functional-connectivity studies further substantiate these observations. Manes et al. (2018) reported that PD patients with speech difficulties exhibit diminished connectivity between the left putamen and the left superior temporal gyrus, a region integral to auditory feedback and speech-error correction [36,57,58,59]. This disruption may relate to reduced capacity to integrate sensory feedback, contributing to hypophonia and articulatory instability. Concurrently, increased connectivity between the left internal globus pallidus and cortical areas such as the dorsal premotor cortex and angular gyrus suggests compensatory reorganisation within cortico-striatal circuits [36,60]. Such adaptations may initially preserve speech performance but could become maladaptive as degeneration progresses.

Collectively, these findings highlight that striatal dopaminergic dysfunction is a key but not exclusive driver of speech difficulties in PD. The persistence of dysarthria despite dopaminergic therapy supports a model in which impaired basal-ganglia output is compounded by cortical dysregulation, altered auditory feedback processing, and diminished sensorimotor integration. This broader network involvement aligns with evidence from transcranial magnetic stimulation studies showing that targeted modulation of temporal and motor cortical regions can improve articulation and speech rhythm [61,62]. Such results underscore the potential for neuromodulatory and behavioural interventions aimed at enhancing compensatory connectivity within the motor-speech system.

### 4.3. CSF Biomarker Profiles Associated with Speech Difficulties

In contrast to the robust associations observed with clinical and dopaminergic imaging measures, cerebrospinal fluid biomarkers did not differentiate early PD patients with and without speech difficulties. This absence of group-level differences suggests that the mechanisms driving speech difficulties in early PD may not be directly captured by the neurochemical markers typically associated with global neurodegeneration.

These findings align with evidence indicating that while CSF biomarkers such as α-synuclein, amyloid-beta, and tau proteins are sensitive indicators of disease progression, including cognitive decline, they may not reflect the subtle neural dysfunctions underlying hypokinetic dysarthria [63]. Previous studies have shown that reduced CSF α-synuclein correlates with motor decline, potentially extending to speech-related neural regions [48], while altered tau and amyloid-beta levels are linked to cognitive impairment affecting executive and linguistic processes essential for speech production [64]. Similarly, pro-inflammatory cytokines such as IL-6 and TNF-α have been associated with worsening motor and non-motor symptoms [65], which could indirectly influence speech performance.

However, the current findings suggest that in the early, clinically mild stages of PD—particularly among treatment-naïve patients—these biomarker alterations may not yet be pronounced enough to differentiate those with and without speech difficulties. This supports the interpretation that early speech difficulties are more likely to arise from localised dopaminergic and motor network dysfunction rather than from widespread proteinopathy or neuroinflammation.

In levodopa-treated patients, the continued absence of significant biomarker differences reinforces this interpretation. While α-synuclein, tau, phosphorylated tau (*p*-tau), and amyloid-beta levels were comparable between groups, previous research indicates that their trajectories evolve over time and may interact with cognitive rather than motor manifestations of PD [20]. Baek et al. (2021) reported that patients with low baseline amyloid-beta levels exhibit faster declines in α-synuclein and accelerated increases in t-tau, *p*-tau, and neurofilament light chain, particularly when cognitive impairment is present [20]. The absence of a similar pattern in the current sample suggests that speech difficulties, when occurring without cognitive decline, may follow a distinct neurobiological course.

### 4.4. Clinical Pharmacology and Treatment Implications

Importantly, the present study was not designed to assess pharmacological treatment effects, treatment responsiveness, or longitudinal changes following dopaminergic therapy. Treatment-naïve and levodopa-treated participants were analysed as independent cross-sectional cohorts and were not longitudinally linked. In addition, detailed information regarding levodopa equivalent daily dose, duration of therapy, or use of adjunctive dopaminergic agents was not available, limiting direct pharmacological interpretation.

Despite these constraints, the persistence of speech difficulties in levodopa-treated patients -together with the strong association between speech impairment, motor severity, and putaminal dopaminergic deficits- is consistent with extensive clinical evidence that speech impairment in PD represents a dopamine-limited symptom domain. Previous studies have demonstrated that hypokinetic dysarthria often shows minimal or inconsistent improvement following dopaminergic therapy, even when limb motor symptoms respond favourably [8,26,66]. This dissociation suggests that while nigrostriatal dopamine depletion contributes to speech motor dysfunction, dopaminergic replacement alone is insufficient to normalise the complex neural networks supporting speech production.

Neuroimaging studies further support this interpretation, indicating that dopaminergic therapy modulates certain cortico-striatal connections involved in speech prosody and motor preparation, but fails to fully restore auditory–motor integration and speech self-monitoring processes [15,53,54]. Functional imaging data suggest that levodopa may facilitate partial compensatory recruitment of premotor and supplementary motor areas, while deficits within temporal auditory regions and cerebellar networks persist [15,36,54,56]. These findings align with the present results, in which speech difficulties remained evident in levodopa-treated patients despite improvements in overall motor performance.

Clinically, these observations underscore the limited efficacy of dopaminergic escalation as a strategy for managing speech impairment in PD. Increasing dopaminergic therapy may expose patients to a higher risk of adverse effects, including dyskinesia and neuropsychiatric complications, without delivering meaningful speech benefit [8,26]. Consequently, speech impairment requires targeted management strategies beyond pharmacological optimisation.

Non-pharmacological interventions, particularly speech and language therapy, remain central to the management of PD-related speech difficulties. Behavioural interventions aimed at improving vocal loudness, articulation, speech timing, and communicative effectiveness have demonstrated benefits independent of dopaminergic status [8,26,66]. Moreover, the limited dopaminergic responsiveness of speech highlights the need for novel therapeutic approaches targeting non-dopaminergic mechanisms, including cortical and cerebellar modulation, sensorimotor integration, and auditory feedback processing [54,61,62].

### 4.5. Limitations and Future Directions

Several limitations should be considered when interpreting the present findings. First, speech impairment was assessed using a single examiner-rated item from the MDS-UPDRS Part III (Item 3.1), which provides a global index of speech impairment but does not capture specific speech subsystems such as phonation, articulation, prosody, or speech rate, nor does it reflect patient-reported communicative participation. As a result, subtle or subsystem-specific speech abnormalities may have been underestimated.

Second, cerebrospinal fluid biomarker data were missing for a proportion of participants, particularly within the levodopa-treated cohort. Although sensitivity analyses were performed, reduced CSF data availability may have limited statistical power to detect modest associations between speech difficulties and neurochemical markers.

Third, levodopa-treated participants were analysed as a single group without stratification by levodopa equivalent daily dose, duration of therapy, or use of adjunctive dopaminergic medications such as dopamine agonists or MAO-B inhibitors. This limits interpretation of treatment-related heterogeneity and precludes conclusions regarding pharmacological responsiveness of speech impairment.

Finally, the cross-sectional design of the study prevents inference regarding causality, disease progression, or longitudinal treatment effects. Longitudinal studies have demonstrated that speech impairment in PD may follow distinct trajectories and may precede or evolve independently of other motor and cognitive symptoms, highlighting the importance of repeated and detailed speech assessment over time [67,68].

Future research should therefore adopt longitudinal and multimodal approaches integrating detailed acoustic speech analysis with advanced neuroimaging techniques, including functional and structural connectivity measures, to better characterise the neural mechanisms underlying speech decline in PD [69,70,71]. In addition, further investigation into cognitive–motor interactions and compensatory network recruitment is warranted, given evidence that executive and temporal processing deficits may exacerbate speech impairment even in the absence of overt dementia [72]. Finally, exploration of targeted non-pharmacological and neuromodulatory interventions may provide novel therapeutic avenues for addressing the multifactorial nature of speech impairment in PD [73,74].

## 5. Conclusions

Speech difficulties in early PD appear to result from the combined effects of ageing, motor severity, and striatal dopaminergic dysfunction. Advancing age reduces neuromuscular control and neural plasticity, while higher MDS-UPDRS Part III scores—reflecting bradykinesia, rigidity, and axial motor deficits—emerge as the strongest predictors of these difficulties. Although cerebrospinal fluid biomarkers showed no significant group differences, reduced [^123^I]FP-CIT uptake in the putamen indicates that early presynaptic dopaminergic loss is associated with speech difficulties. The persistence of speech difficulties in levodopa-treated patients suggests additional non-dopaminergic contributions involving disrupted cortico-striatal and auditory feedback networks. These associations should be interpreted within the context of a cross-sectional design and do not indicate causal mechanisms or pharmacological treatment effects. Together, these findings indicate that speech difficulties are an early and sensitive marker of network-level dysfunction in PD, highlighting the importance of early identification and the need for longitudinal, multimodal research to inform targeted therapeutic interventions.

## Figures and Tables

**Table 1:** Demographic characteristics, Cognitive performance, Clinical characteristics, CSF biomarkers and [^123^I]FP-CIT SBR in early PD treatment-naïve patients with and without speech difficulties. SBR = specific binding ratio.

	Early PD Treatment-Naïve Patients with Speech Difficulties (N = 167)	Early PD Treatment-Naïve Patients Without Speech Difficulties (N = 209)	*p* Value
**Demographic Characteristics**			
Age (years) [mean (±SD)]	63.78 (±8.8)	60.33 (±9.9)	**<0.001**
Sex [mean (±SD)]	0.71 (±0.4)	0.61 (±0.4)	**0.04**
Disease duration (months) [mean (±SD)]	6.63 (±6.1)	6.65 (±6.8)	0.98
**Cognitive Performance**			
MoCA [mean (±SD)]	26.98 (±2.3)	27.29 (±2.3)	0.20
**Clinical Characteristics**			
MDS-UPDRS Part I [mean (±SD)]	6.13 (±3.9)	5.04 (±4)	**0.01**
MDS-UPDRS Part II [mean (±SD)]	7.16 (±4.5)	4.94 (±3.6)	**<0.001**
MDS-UPDRS Part III [mean (±SD)]	24.14 (±8.5)	18.47 (±8.2)	**<0.001**
MDS-UPDRS Part III(ON-state) [mean (±SD)]	24.14 (±8.5)	18.47 (±8.2)	**<0.001**
MDS-UPDRS Part IV [mean (±SD)] ^1^	0	0	-
MDS-UPDRS Total [mean (±SD)]	37.42 (±13)	28.45 (±12)	**<0.001**
MDS-UPDRS Total (ON-state) [mean (±SD)]	37.42 (±13)	28.45 (±12)	**<0.001**
Holen & Yard (ON-state) [mean (±SD)]	1.67 (±0.47)	1.49 (±0.50)	**<0.001**
^1^ t cannot be computed because at least one group contained no observations.			
CSF Biomarkers *			
abeta [mean (±SD)]	829.01 (±296.1) ^a^	830.55 (±288.3) ^b^	0.96
tau [mean (±SD)]	173.12 (±61.8) ^c^	165.80 (±53) ^d^	0.23
ptau [mean (±SD)]	15.23 (±5.7) ^e^	14.49 (±4.8) ^f^	0.20
asyn [mean (±SD)]	1536.85 (±711.1) ^d^	1495.23 (±636.1) ^g^	0.55
* Unavailable data’s ratio (%): ^a^ 12%, ^b^ 9%, ^c^ 5%, ^d^ 4%, ^e^ 11%, ^f^ 10%, ^g^ <1%			
**[^123^I]FP-CIT SBR**			
contralateral_caudate [mean (±SD)]	1.71 (±0.5)	1.88 (±0.5)	**0.002**
ipsilateral_caudate [mean (±SD)]	1.97 (±0.5)	2.24 (±0.5)	**<0.001**
mean_caudate [mean (±SD)]	1.84 (±1.8)	2.06 (±0.5)	**<0.001**
contralateral _putamen [mean (±SD)]	0.63 (±0.2)	0.72 (±0.2)	**<0.001**
ipsilateral _putamen [mean (±SD)]	0.83 (±0.2)	1.03 (±0.3)	**<0.001**
mean_putamen [mean (±SD)]	0.73 (±0.2)	0.87 (±0.2)	**<0.001**
contralateral _striatum [mean (±SD)]	2.34 (±0.6)	2.60 (±0.7)	**<0.001**
ipsilateral _striatum [mean (±SD)]	2.81 (±0.8)	3.27 (±0.8)	**<0.001**
mean_striatum [mean (±SD)]	1.29 (±0.3)	1.46 (±0.3)	**<0.001**

**Table 2:** Multivariate Binary Logistic Regression analysis of predictor variables on early PD treatment-naïve patients with and without speech difficulties.

	B	S.E.	Wald	df	Sig.	Exp(B)
**Age**	0.027	0.012	4.836	1	**0.028**	1.028
**Sex at birth (1)**	0.388	0.239	2.649	1	0.104	1.474
**MDS-UPDRS part III ON**	0.071	0.016	18.704	1	**<0.001**	1.074
**Hoehn and Yahr ON (1)**	−0.237	0.278	0.728	1	0.393	0.789
**mean_putamen**	−1.620	0.483	11.257	1	**<0.001**	0.198

Sex at birth (1) was coded as a binary variable (0 = female, 1 = male). Hoehn and Yahr ON (1) indicates category 1 of the Hoehn and Yahr staging scale assessed in the ON medication state.

**Table 3:** Demographic characteristics, Cognitive performance, MDS-UPDRS Scales, CSF biomarkers and [^123^I]FP-CIT SBR in early PD levodopa-treated patients with and without speech difficulties. SBR = specific binding ratio.

	Early PD Levodopa-Treated Patients with Speech Difficulties (N = 76)	Early PD Levodopa-Treated Patients Without Speech Difficulties (N = 57)	*p* Value
**Demographic Characteristics**			
Age (years) [mean (±SD)]	63.53 (±7.9)	60.63 (±10)	0.66
Sex [mean (±SD)]	0.71 (±0.4)	0.63 (±0.4)	0.33
Disease duration (months) [mean (±SD)]	5.82 (±5.9)	4.7 (±6.2)	0.33
**Cognitive Performance**			
MoCA [mean (±SD)]	25.70 (±3.5)	26.25 (±3.3)	0.36
Clinical Characteristics *			
MDS-UPDRS Part I [mean (±SD)]	7.95 (±5.4)	6.40 (±4.3)	0.08
MDS-UPDRS Part II [mean (±SD)]	8.72 (±5.3)	6.23 (±3.7)	**0.003**
MDS-UPDRS Part III [mean (±SD)]	29.26 (±10.3) ^a^	21.94 (±8.3) ^b^	**<0.001**
MDS-UPDRS Part III(ON-state) [mean (±SD)]	24.03 (±9.9) ^c^	17.02 (±8.1) ^d^	**<0.001**
MDS-UPDRS Part IV [mean (±SD)]	0.05 (±0.2) ^e^	0.0 (±0.0)	0.15
MDS-UPDRS Total [mean (±SD)]	45.63 (±15) ^a^	34.03 (±12.6) ^b^	**<0.001**
MDS-UPDRS Total (ON-state) [mean (±SD)]	40.55 (±15.6) ^c^	29.63 (±11.3) ^d^	**<0.001**
Holen & Yard (ON-state) [mean (±SD)]	1.81 (±0.46) ^c^	1.63 (±0.48) ^d^	**0.045**
* Unavailable data’s ratio (%): ^a^ 18%, ^b^ 45%, ^c^ 11%, ^d^ 5%, ^e^ 1%			
CSF Biomarkers *			
abeta [mean (±SD)]	734.39 (±293) ^a^	788.78 (±261.1) ^b^	0.36
tau [mean (±SD)]	169.01 (±68.5) ^c^	174.44 (±75.7) ^d^	0.70
ptau [mean (±SD)]	14.55 (±5.8) ^a^	15.87 (±7.6) ^e^	0.34
asyn [mean (±SD)]	1425.80 (±623.2) ^c^	1463.12 (±660) ^f^	0.77
* Unavailable data’s ratio (%): ^a^ 23%, ^b^ 35%, ^c^ 14%, ^d^ 29%, ^e^ 38%, ^f^ 28%			
[^123^I]FP-CIT SBR *			
contralateral_caudate [mean (±SD)]	1.39 (±0.4)	1.58 (±0.4) ^a^	**0.02**
ipsilateral_caudate [mean (±SD)]	1.67 (±0.5)	1.88 (±0.5) ^a^	**0.02**
mean_caudate [mean (±SD)]	1.53 (±0.4)	1.73 (±0.4) ^a^	**0.01**
contralateral _putamen [mean (±SD)]	0.51 (±0.1)	0.60 (±0.1) ^a^	**0.003**
ipsilateral _putamen [mean (±SD)]	0.66 (±0.2)	0.78 (±0.2) ^a^	**0.008**
mean_putamen [mean (±SD)]	0.58 (±0.1)	0.69 (±0.1) ^a^	**0.002**
contralateral _striatum [mean (±SD)]	1.90 (±0.5)	2.18 (±0.6) ^a^	**0.008**
ipsilateral _striatum [mean (±SD)]	2.33 (±0.7)	2.66 (±0.7) ^a^	**0.01**
mean_striatum [mean (±SD)]	1.06 (±0.3)	1.21 (±0.3 ) ^a^	**0.007**
* Unavailable data’s ratio (%): ^a^ 1%			

**Table 4:** Multivariate Binary Logistic Regression analysis of predictor variables on early PD levodopa-treated patients with and without speech difficulties.

	B	S.E.	Wald	df	Sig.	Exp(B)
**Hoehn and Yahr ON**			0.582	2	0.748	
**Hoehn and Yahr ON (1)**	−0.390	0.511	0.582	1	0.446	0.677
**Hoehn and Yahr ON (2)**	19.335	28,024.390	0.000	1	0.999	249,410,221.953
**MDS-UPDRS part II**	0.014	0.053	0.073	1	0.788	1.014
**MDS-UPDRS part III ON**	0.079	0.028	7.913	1	**0.005**	1.082
**mean_putamen**	−2.405	1.098	4.801	1	**0.028**	0.090

Hoehn and Yahr ON (1) indicates stage 1 of the Hoehn and Yahr scale assessed in the ON medication state. Hoehn and Yahr ON (2) indicates stage 2 of the Hoehn and Yahr scale assessed in the ON medication state.

## Data Availability

The data supporting this study are available from the Parkinson’s Progression Markers Initiative (PPMI) database (www.ppmi-info.org). Access to the dataset is granted to qualified researchers upon registration and data use agreement approval through the PPMI Data Access Committee.

## Abbreviations

The following abbreviations are used in this manuscript:

CSF	Cerebrospinal fluid
fMRI	Functional magnetic resonance imaging
MDS-UPDRS	Movement Disorder Society—Unified Parkinson’s Disease Rating Scale
MoCA	Montreal Cognitive Assessment
PD	Parkinson’s disease
PPMI	Parkinson’s Progression Markers Initiative
SBR	Specific binding ratio
SPECT	Single-photon emission computed tomography
SMA	Supplementary motor area
